# PD-L1/PD-1 Axis in Multiple Myeloma Microenvironment and a Possible Link with CD38-Mediated Immune-Suppression

**DOI:** 10.3390/cancers13020164

**Published:** 2021-01-06

**Authors:** Federica Costa, Valentina Marchica, Paola Storti, Fabio Malavasi, Nicola Giuliani

**Affiliations:** 1 Department of Medicine and Surgery, University of Parma, 43126 Parma, Italy; federica.costa@unipr.it (F.C.); valentina.marchica@unipr.it (V.M.); paola.storti@unipr.it (P.S.); 2 Department of Medical Science, University of Turin and Fondazione Ricerca Molinette, 10123 Turin, Italy; 3 Hematology, Azienda Ospedaliero-Universitaria di Parma, 43126 Parma, Italy

**Keywords:** PD-L1, PD-1, multiple myeloma, microenvironment, CD38, adenosine

## Abstract

**Simple Summary:**

Despite the impressive clinical impact of programmed death-ligand 1 (PD-L1)/programmed cell death-1 (PD-1) blockade in solid tumors, the use of these checkpoint inhibitors in multiple myeloma (MM) still remains debated with unsatisfying clinically meaningful results. In this review we summarize the available literature data on the PD-L1/PD-1 expression profile, highlighting some discrepancies and providing a rationale for the combination with anti-CD38 antibodies, in light of the immunosuppressive effect of CD38-mediated adenosine production.

**Abstract:**

The emerging role of the PD-1/PD-L1 axis in MM immune-microenvironment has been highlighted by several studies. However, discordant data have been reported on PD-1/PD-L1 distribution within the bone marrow (BM) microenvironment of patients with monoclonal gammopathies. In addition, the efficacy of PD-1/PD-L1 blockade as a therapeutic strategy to reverse myeloma immune suppression and inhibit myeloma cell survival still remains unknown. Recent data suggest that, among the potential mechanisms behind the lack of responsiveness or resistance to anti-PD-L1/PD-1 antibodies, the CD38 metabolic pathways involving the immune-suppressive factor, adenosine, could play an important role. This review summarizes the available data on PD-1/PD-L1 expression in patients with MM, reporting the main mechanisms of regulation of PD-1/PD-L1 axis. The possible link between the CD38 and PD-1/PD-L1 pathways is also reported, highlighting the rationale for the potential use of a combined therapeutic approach with CD38 blocking agents and anti-PD-1/PD-L1 antibodies in order to improve their anti-tumoral effect in MM patients.

## 1. Introduction

Immune dysfunction is one of the hallmarks of multiple myeloma (MM). The interaction between MM cells and bone marrow (BM) microenvironment cells, together with hypoxic condition, create a permissive niche which supports immune escape and tumor growth, through the production of several factors including transforming growth factor (TGF)-β, interleukin (IL)-10, IL-6, and prostaglandin E2, known to have immunosuppressive properties. Specifically, the MM niche is characterized by impaired dendritic cell (DC) differentiation and maturation, high levels of myeloid derived suppressor cells (MDSCs) and regulatory T cells (Tregs), along with an unbalanced ratio of T helper (Th)1/Th2 cells and altered natural killer (NK) cell cytotoxic activity [1]. Moreover, CD8+ T cells exhibit exhaustion markers such as programmed cell death-1 (PD-1), cytotoxic T lymphocyte antigen-4 (CTLA-4), T cell immunoglobulin-3 (TIM-3), and lymphocyte-activation gene 3 (LAG3), and high levels of the senescence markers killer-cell lectin like receptor G1 (KLRG1), CD57 and CD160, resulting in low responsiveness [2]. A Th17 polarization is also observed in MM patients with consequent increased of IL-17 levels in BM and peripheral blood (PB) of MM patients, which promotes tumor proliferation and induces bone lesions [3]. Targeting the immune-microenvironment thus represents an effective strategy to prevent tumor progression.

## 2. The Role of Programmed Death-Ligand 1 (PD-L1)/Programmed Cell Death-1 (PD-1) Axis in Cancer

In the last few years, the role of immune checkpoints as efficient therapeutic targets has emerged in different solid tumors (melanoma, non-small cell lung cancer, renal cell, carcinoma, head and neck carcinoma) and Hodgkin’s lymphoma [4,5,6,7]. Immune checkpoints constitute a family of both inhibitory and stimulatory molecules, critical for the maintenance of self-tolerance and the regulation of immune responses. Several stimulatory signals, such as CD28/CD80, CD86, CD27/CD70, CD40/CD40L, ICOS/ICOSL, are involved in the activation and expansion of T cells; on the other hand, inhibitory pathways are similarly important to prevent autoimmunity, such as PD-1/PD-L1 and PD-L2, CTLA-4/CD80 and CD86, A2AR/adenosine, LAG3/Major histocompatibility complex (MHC) class II [8,9]. However, tumor cells increase the expression of ligands of the checkpoint receptors as an escape mechanism to immune responses, thus making checkpoint blockade by monoclonal antibodies (mAbs) a new therapeutic strategy for cancer. In this review, we will focus on the PD-L1/PD-1 axis and its involvement in MM immune dysregulation.

PD-1 is a 288 amino acid type I transmembrane protein, part of the CD28 receptor family. PD-1 consists of an Ig-V like extracellular domain, a transmembrane domain, and a cytoplasmic domain that harbors two tyrosine-based signaling motifs. Phosphorylation of the cytoplasmic immune-receptor tyrosine-based inhibitory motif (ITIM) and the immune-receptor tyrosine-based switch motif (ITSM) by Src family kinases [10]. PD-1 is encoded on chromosome 2 and it is mainly expressed on activated/exhausted T and B cells [11,12].

PD-L1, also known as B7-H1 and CD274, is a 40kD Type I transmembrane glycoprotein, which contains an immunoglobulin (Ig)-V and Ig-C-like extracellular domain, a transmembrane domain, and a short cytoplasmic tail that does not contain canonical signaling motifs [13]. PD-L1 is encoded by chromosome 9, constitutively expressed at low levels by cells of the myeloid lineage, including macrophages and DCs, as well as vascular endothelial cells, pancreatic islet cells, and in sites of immune privilege (placenta, testes, eye). The expression of PD-L2, the other ligand of PD-1, is more restricted on DCs and macrophages after activation [14]. Several studies focused on the regulation of PD-L1 expression, which seems to be mediated by both intrinsic and extrinsic mechanisms, in light of its restricted protein expression and the ubiquity of mRNA. The first include epigenetic and post-transcriptional modifications i.e., deacetylation and microRNAs regulations, which decrease PD-L1 expression on tumor cells [15,16,17]. The extrinsic mechanisms are mediated by several pro-inflammatory cytokines, including the most potent interferon gamma (IFN-γ). This factor induces PD-L1 expression at post-transcriptional level, and also binds two sites (200- and 320-base-pair upstream of transcriptional start site) on interferon regulatory factor 1 in PD-L1 promoter [18].

Concerning the functional activity, the engagement of the PD-1 receptor with its ligands PD-L1 or PD-L2 activates PD-1 downstream from Src-homology 2-containing tyrosine phosphatase (SHP-2) and dephosphorylates ZAP70 which leads to the inhibition of T cell proliferation, survival and cytokine production, induces T-cell exhaustion, enhances Tregs development, and decreases NK cell cytotoxicity, granule exocytosis and IFN-γ secretion, through the interference with Protein kinase C (PKC)-θ, PhosphatidylInositol 3-Kinase (PI3K), extracellular signal-regulated kinase (ERK) and AKT activation [19,20]. Of note, PD-L1 also interacts with CD80 expressed on T cells, thus inhibiting their proliferation [21]. More recently, a study from Bar et al. [22] revealed that in vitro PD-L1 blockade increases both monocyte-derived DC differentiation and CD40L-driven DC maturation in healthy donors (HDs). It also enhances the secretion of several inflammatory cytokines as IL-6, IL-8, tumor necrosis factor (TNF)-α, and IL-1β. These effects were specifically observed with PD-L1 blockade but not with anti-PD-1 mAbs, thus suggesting an additional role for PD-L1 as a checkpoint for regulating inflammatory phenotype of myeloid cells and antigen presentation in DCs, confirmed by in vivo data [22].

Besides this activity on effector immune cells, PD-L1 also delivers an intrinsic anti-apoptotic intracellular signal in cancer cells, thus conferring resistance to T cell-mediated death without relying on the PD-1-dependent inhibition of T cells [23]. However, how these emerging pro-survival signals are conveyed intracellularly from cell surface PD-L1 is largely unknown. In some tumor cells, this has been shown to stimulate cancer initiation, epithelial to mesenchymal transition (EMT), invasion and metastasis, promote drug resistance and regulate glucose metabolism. A study by Chang et al. described the PD-L1/PD-1 axis involvement in the metabolic competition between tumor cells and tumor-infiltrating T lymphocytes [24]. Specifically, the authors used an established sarcoma mouse model of regressing (R) and progressing (P) tumors [25,26] to test the effect of PD-1 and PD-L1 blockade on tumor/tumor infiltrating lymphocytes (TILs) metabolism. Experimental data confirmed that the extracellular acidification rate (ECAR), an indicator of aerobic glycolysis, was higher in the P tumor and inversely proportional to the metabolism of TILs isolated from that tumor, suggesting that a more aggressive tumor consumes more glucose and limits its availability in the microenvironment. In addition, TILs in the P tumors were PD-1^hi^, consistent with their hypo-responsiveness. Interestingly, the use of anti PD-1 antibodies increased ECAR and oxygen consumption rate (OCR) in P-TILs to levels equal or above those observed in R-TILs, indicating that the treatment reinvigorates the metabolic fitness of the TILs. PD-L1 antibodies, however, primarily promoted aerobic glycolysis, rather than OCR, in the TILs [24].

It is also known that PD-L1 expression stimulates glycolysis and Akt/mammalian target of rapamycin (mTOR) activation in tumor cells while suppressing this pathway in the T cell counterpart. The engagement of PD-1 on activated T cells thus promotes fatty acid β-oxidation of endogenous lipids, in place of glycolysis or amino acid metabolism [27,28]. This event then polarizes T cells towards a regulatory and exhausted phenotype [29]. In line with this evidence, the treatment with PD-L1 blocking antibodies suppressed tumor progression and glucose uptake in tumor cells while enhancing mTOR activity of T cells [24]. The activation of Akt/mTOR signaling is further promoted by the hypoxia-inducible transcription factor (HIF-1α) which additionally sustains fatty acid and protein synthesis to support malignant cell survival [30].

The number of Food and Drug Administration (FDA)-approved agents blocking PD-L1/PD-1 axis is rapidly enlarging with indications for treatment of a broad spectrum of malignancies, ranging from classical Hodgkin lymphoma to head and neck squamous cell carcinoma (HNSCC), melanoma and urothelial cancers, both as monotherapy and in combination with other agents. More recently, the use of the anti-PD-1, pembrolizumab, has been approved for the treatment of all solid tumors with tumor mutational burden (TMB) equal to or greater than 10 mutations/megabase as measured by the FoundationOne CDx assay. High TMB cancers tend to have more immunogenic neoantigens (“FDA approves third oncology drug that targets a key genetic driver of cancer, rather than a specific type of tumor, in 2019”) and recognition of tumor neoantigens by host T cells is one of the critical factors predicting immunotherapy response [31]. However, a good response to anti-PD-1 antibody has been detected even in tumors with relatively low mutational burden, suggesting that mutation quality is more important than mutation quantity. Moreover, response to PD-1 inhibitors differs between inflamed and non-inflamed tumors [32] and cancer stemness and intra-tumoral heterogeneity may have a greater impact on immune response and may better predict immunotherapy outcomes than TMB [33]. Overall, these data thus underline the need to identify and validate other biomarkers of sensitivity and resistance to this class of agents.

## 3. PD-L1/PD-1 Distribution in MM Microenvironment

Among hematological malignancies, the role of PD-L1/PD-1 axis in MM is still debated. The expression profile of PD-L1/PD-1 axis in MM has been investigated by numerous research groups; however, the use of mAbs blocking this pathway is still under discussion, at least in part, because of discordant data on PD-L1/PD-1 distribution on malignant plasmacells (PCs) or immune effector cells within the BM microenvironment.

In vitro studies on MM models revealed that PD-L1 is expressed by PCs and, as in the other tumors, PD-L1+ PCs inhibit cytotoxic T cell lymphocyte (CTL) activities, thus contributing to the immune escape. Studies on human myeloma cell lines (HMCLs) discovered that CD138+PD-L1+ cells show a more aggressive phenotype, with increased proliferation rate and resistance to conventional anti-MM therapies, as dexamethasone, melphalan and bortezomib, mediated by the activation of PI3K/AKT signaling pathway [34,35]. The expression of cell cycle-related genes, CCND3 and CDK6, and anti-apoptotic markers, BCL2 and MCL1, was also upregulated in PD-L1+ myeloma cells [35].

It has been demonstrated that the cross-talk between MM cells and BM stromal cells (MSCs) promotes tumor survival, by suppressing CD4+ T cells activity through the PD-L1/PD-1 axis [36]. Specifically, the authors described that PD-L1 shRNA in BM MSCs effectively reversed BM MSCs-mediated inhibition of IFN-γ, and stimulation of IL-4 and TGF-β production in CD4+ T cells, thus reversing the reduction of Th1/Th2 and Th17/Treg. In addition, BM MSCs promotion of 5TGM1 cell proliferation was inhibited after PD-L1 silencing, which suggested a role of PD-L1 in BM MSCs-induced MM growth [36]. Furthermore, BM MSCs induce PD-L1 expression on MM cells, generating an aggressive phenotype [34].

On the other hand, results from ex vivo studies show many discrepancies. Several groups described that PD-L1 expression is limited to PCs (evaluated as CD138+/CD38+ cells) from MM patients and is absent on those from HDs [34,37,38,39]. In addition, PD-L1 expression was reported to be higher in MM PCs as compared with monoclonal gammopathies of undetermined significance (MGUS) [34,37]. Conversely, other research groups showed no differences in PC PD-L1 expression among MM, MGUS and HDs [40,41,42]. Few data are currently available on smoldering MM (SMM) patients. A study from Dhodapkar et al. [43] interestingly revealed that high PD-L1 expression on PCs was associated with disease progression in patients with MGUS and asymptomatic MM. Similarly, a minor study on bone biopsies showed that PD-L1 on PCs increased from SMM diagnosis to the onset of active MM after 2 years [44]. The association between the expression of PD-L1 and the prognosis of tumors was also described in a recent study from Lee BH et al. [45] who developed a prognostic nomogram, finding that a combination of PD-L1 expression in PCs evaluated by the quantitative immunofluorescence (QIF) method, and clinical parameters (age, cytogenetics, and lactate dehydrogenase) effectively predicted poor prognosis in newly diagnosed MM. In addition, a recent study from our group described that SMM and active MM patients share a similar PD-L1/PD-1 BM immune profile [46]. Moreover, the PD-L1/PD-1 axis could be involved in the development of clonal resistance as demonstrated by PD-L1 high levels in relapsed or refractory MM patients [34]. Furthermore, Paiva et al. [40] highlighted PD-L1 upregulation in patients with minimal residual disease (MRD), suggesting that residual PD-L1+ myeloma cells have an increased ability to survive and escape immunosurveillance.

It is important to note that most of the studies analyzed PD-L1 using a basic flow panel to detect PCs (CD138+/CD38+ cells), with only one study focused on PC clonality established with κ/λ staining [43], which did not show any differences between clonal and non-clonal PCs. Two studies [40,41] used a more extended panel with CD45/CD19/CD56 to distinguish and analyze only PCs with aberrant phenotype. These methodological differences could in part explain the discrepancies of literature data on PD-L1 expression. Furthermore, the majority of the studies reported high heterogeneity in PD-L1 expression among patients with the same stage of disease, which makes more difficult to select the best subset of patients who could benefit from PD-L1/PD-1 blockade. 

To our knowledge, only one study found that PD-L1 is expressed at higher levels in hyperdiploid patients, in line with its localization on chromosome 9 [39]. No other study investigated a correlation between PD-L1 expression and other cytogenetic abnormalities.

PD-L1 expression was also detected by immunohistochemistry on PCs from patients with extra-medullary disease, together with PD-1+ T cells infiltrating the extra-medullary lesions, suggesting a possible link between the PD-L1/PD-1 axis and a poor prognosis [47]. However, further studies are needed to clarify the involvement of this checkpoint in the onset of extra-medullary disease.

Within MM BM microenvironment, PD-L1 is also expressed by myeloid cells, including monocytes, DCs and MDSCs. A study from Ray et al. [38] revealed that plasmacytoid DCs (pDCs), which play an important role in MM cell growth and prolonged survival, express PD-L1 at higher levels as compared with MM PCs, and the blockade of PD-L1/PD-1 interactions between pDC–T cell/NK cells inhibits MM proliferation. Moreover, PD-L1+ DCs are mainly localized in the BM of MM patients, while a small fraction can be detected in the PB, as for myeloid CD141+ DCs which are positively correlated with PD-L1+ PCs % [48]. PD-L1+ DC diminished ability to trigger T cell response was also proved to contribute to immune dysfunction [49]. More than DCs, MDSCs, specifically myeloid MDSCs in newly diagnosed MM (NDMM) and granulocytic MDSCs in relapsed MM (RMM), seem to express PD-L1 at high levels, as demonstrated by Gorgun et al. [39]. However, a study from Castella et al showed no differences in terms of total PD-L1+ MDSCs % among NDMM, RMM and MM patients in remission [50]. On the other hand, very limited data are currently available on PD-L1 expression by MDSCs in patients with asymptomatic myeloma. Recently, Dhodapkar’s group, using single-cell mass cytometry analysis of bone marrow mononuclear cells, found that PD-L1 was increased in the myeloid compartment of MGUS and MM as compared with HDs; however, no differences were reported between MGUS and MM patients [51].

A study from An et al. [52] has also described PD-L1 up-regulation during in vitro osteoclastogenesis, suggesting an immune-suppressive function of osteoclasts (OCs) in myeloma microenvironment. OCs in turn induced PD-L1 expression in MM cell lines, via an APRIL-dependent manner, thus providing additional immune inhibition by OCs [52].

Focusing on PD-1 distribution, several studies described higher PD-1 expression levels on T cells from MM patients as compared with HDs, accompanied by a loss of function, on both circulating T cells and BM CD8+ T and NK cells [39,53,54]. PD-1 expression was also correlated with T cell exhaustion/senescence in MM patients [55]. Conversely, Paiva et al. [40] showed no differences among MM, MGUS and HDs on both T cells and NK cells; while a significant increase in PD-1 expression on both CD4+ and CD8+ cells was detected in MRD+ and RMM patients as compared with NDMM. In contrast with these results, a study from Kwon M et al. [56] compared %CD8+PD-1+ cells between MGUS/SMM and NDMM which displayed a higher % as compared with the other group. In addition, PD-1 expression has been described on the anergic BM Vg9Vd2 T cell subset from MGUS patients and remained upregulated in MM after clinical remission [50].

To conclude, high discrepancies characterize the current scientific literature on PD-L1/PD-1 distribution in MM, making it difficult to evaluate which patient subset could better benefit from PD-L1/PD-1 blockade. Table 1 summarizes the current literature data in patients with monoclonal gammopathies.

A soluble form of PD-L1 has been also detected in BM plasma of MM patients. Specifically, the way in which PD-L1 soluble levels predict treatment response and progression-free survival (PFS) in newly diagnosed MM patients has been described [57]; moreover, high PD-L1 soluble levels have been associated with shorter overall survival (OS) rates and worse responses after autologous stem cell transplantation (ASCT) in MM patients [58]. No statistical correlation was found between PD-L1 soluble levels and cytogenetic risk. However, the mechanisms that generate soluble PD-L1 remain poorly understood.

## 4. Mechanisms of PD-L1 Regulation in MM

Numerous studies have analyzed the possible mechanisms behind the regulation of PD-L1 expression in MM. 

As for other cancers, a role of IFN-γ has been described which up-regulates PD-L1 expression via IRF1 [18]. IFN-γ produced by BM microenvironment cells, as activated Th1, macrophages, NK and natural killer T (NKT) cells, activates Janus kinase/signal transducers and activators of transcription (JAK/STAT) and mitogen-activated protein kinase kinase (MEK)/ERK pathways, strongly inducing PD-L1 expression [37]. Of note, STAT1 activation is also stimulated by toll like receptor (TLR), as TLR2, TLR4, TRL7 and TLR9, highly expressed by MM cells [59], through the MyD88/TRAF6 pathway. Interestingly, the inhibition of the MyD88 and TRAF6 adaptor proteins of the TLR pathway blocked not only PD-L1 expression induced by TLR ligands but also that mediated by IFN-γ [37]. Other mechanisms mediated by phosphatase and tensin homolog deleted on the chromosome ten (PTEN)/PI3K/AKT/mTOR pathway have been described in solid tumors, where loss of PTEN promoted cell proliferation, cell invasion and significant increase in the levels of phospho-AKT and phospho-mTOR, resulting in enhanced protein translation of PD-L1 [15,60,61].

Within the myeloma microenvironment, BM MSCs enhance PC proliferation and survival, via both cell-to-cell contact and the release of soluble factors. BM MSCs are also involved in PD-L1 up-regulation on MM cells [34]. This effect is mediated by IL-6 BM MSC release which activates JAK2, STAT3 and the MEK1/2 pathway [34]. In line with this, PD-L1 down-regulation was observed in MM cells, after treatment with the JAK inhibitor ruxolitinib [62].

Another mechanism of PD-L1 up-regulation in MM is the binding between the proliferation-inducing ligand (APRIL), secreted by eosinophils, OCs and myeloid cells, and B-cell maturation antigens (BCMAs) on MM cells. This interaction leads to MEK1/2 phosphorylation which further induces PD-L1 up-regulation in MM [52,63].

Several studies revealed the impact of different conventional anti-MM therapy on PD-L1 expression. Specifically, it has been demonstrated that immunomodulatory drugs (IMiDs), except for thalidomide, induce PD-L1 expression on IMiDs-resistant HMCLs and primary PCs from relapsed/refractory MM patients (RR-MM). This effect was mediated by the BCMA–APRIL pathway. In fact, IMiDs induce APRIL expression, known to up-regulate PD-L1 expression as mentioned above, through Ikaros degradation in MM cells [64]. Conversely, an in-vitro study on primary cells from RR-MM treated with lenalidomide revealed that the compound modestly decreased PD-L1 surface expression on malignant PCs, and more significantly on monocytes/macrophages and myeloid MDSCs. In addition, both lenalidomide and pomalidomide in vitro treatment significantly reduced PD-1 surface expression on CD4+ and CD8+ T cells, and NK cells [39,65] (Table 2).

Proteasome inhibitors bortezomib, carfilzomib, and ixazomib also affect PD-L1 levels in MM, by inducing its up-regulation [63]. Moreover, a study by Ray et al. described an increased PD-L1 expression on MM cells after treatment with histone deacetylase inhibitors (HDACis) [66]. Finally, Stocker et al demonstrated that PD-L1 expression increases on monocytes, myeloid and pDCs during treatment with bortezomib-thalidomide-dexamethasone (VTD); on the other hand, daratumumab prevents this effect [67].

Together all these data thus provide a rationale for treatment combinations, in order to increase the clinical activity of PD-L1/PD-1 blockade in MM. However, clinical trials using combinations of anti-PD-1/PD-L1 mAbs and IMiDs in MM were put on hold by the FDA because of severe adverse events; while combinations of HDACi are only available for patients with advanced melanoma (NCT02935790 and NCT02032810). A phase I clinical trial evaluated the effect of pembrolizumab in combination with standard of care treatments, including lenalidomide and carfilzomib, in MM patients (MK-3475-023/KEYNOTE-023). However, there are no currently available results from a pembrolizumab-combination with the proteasome inhibitor.

## 5. The Immune Suppressive Role of PD-L1/PD-1 Axis in MM Microenvironment: Preclinical and Clinical Evidence 

**Figure 1:**
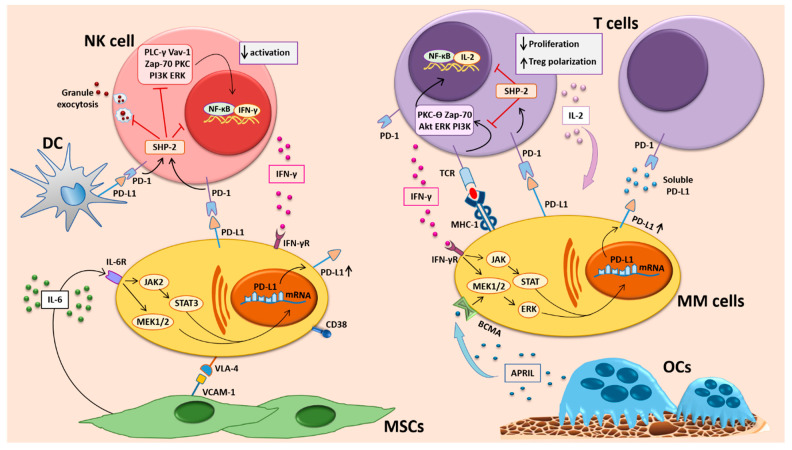
PD-L1/PD-1 distribution and mechanisms of regulation in the MM BM niche. Abbreviations: DC, dendritic cell; MSCs, mesenchymal stromal cells; MM, multiple myeloma; NK, natural killer; OCs, osteoclasts.

Pre-clinical studies on PD-L1/PD-1 blockade in MM provided very promising results. Indeed, in vitro PD-L1/PD-1 blockade overcame BM MSC-mediated MM growth and directly enhanced NK and T cell mediated anti–MM responses [39,53]. As in the other tumors, PD-L1 expressing MM cells can inhibit the activity of CTLs, acquiring a proliferative advantage which results in immune evasion and resistance to anti-myeloma agents, compared with PD-L1–negative myeloma cells [34]. In addition, PD-L1+ pDCs capacity to induce cytotoxic activity of T cells and NK cells against MM PCs was restored after treatment with PD-L1/PD-1 blocking mAbs [38]. In vivo experiments on the 5T33 murine MM models showed that PD-L1 blockade prolonged mice survival after autologous (syngeneic) stem-cell transplantation plus administration of a cell-based vaccine or after irradiation [54,67,68]. PD-1 blockade also prolonged the survival in disseminated myeloma-bearing mice [67,68], by mainly acting on CD4+ or CD8+ T cells [67]. In these models, PD-1 expression on both CD8+ and CD4+ T cells was higher in mice with advanced MM as compared to non-tumor bearing ones; moreover, it was found a correlation between the tumor burden and the percentages of PD-1+ T cells, which were defective for the production of pro-inflammatory cytokines (IFN-γ and IL-2) after in vitro stimulation. In addition, these cells expressed increased levels of the exhausted T cell marker, TIM-3 [68]. Moreover, Gorgun et al. demonstrated that lenalidomide treatment enhances the cytotoxic effects of PD-L1/PD-1 blockade in RR-MM [39]. Overall, these studies thus suggested that PD-L1/PD-1 blockade may be an effective therapeutic strategy against MM, both alone and in association with other anti-MM therapeutic strategies. Figure 1 summarizes PD-L1/PD-1 distribution in the MM BM niche and the mechanisms of PD-L1/PD-1 regulation.

Among the different combination treatments, several phase III trials with pembrolizumab in combination with IMiDs were designed and achieved a 44% or 60% overall response rate (ORR) in RR-MM patients. However, the FDA put them on hold in 2017 because of higher incidence of adverse events including neutropenic sepsis, myocarditis and Stevens–Johnson syndrome, which might be associated with an excessive autoimmune reaction [69]. Nevertheless, results from KEYNOTE 183 (pomalidomide + dexamethasone + pembrolizumab) and KEYNOTE 185 (lenalidomide + dexamethasone + pembrolizumab) suggested that anti PD-1 mAbs are more effective in patients with activation of the immune system as MMD; however, it remains to clarify which is the best combination, dose and regimen to avoid the toxicity and increase the anti-tumor effect of these class of agents.

## 6. CD38 and Its Role in MM Microenvironment

In recent studies, the emerging role of CD38 in the biology of MM and as a therapeutic target has been highlighted. CD38 is a 45-kDa type II transmembrane glycoprotein, which plays a dual role as a receptor and ectoenzyme [70]. CD38 is highly expressed in myeloma PCs [71] and activated T cells and NK cells [72]. CD38 is involved in T cell activation and proliferation, B cell differentiation, and neutrophils chemotaxis [70]. In addition, IFN-γ up-regulates CD38 expression on monocytes and plays a specific role in their activation and adhesion processes [73]. A study by our group [74] showed that CD38 is expressed on the surface of early OC progenitors but it is lost during in vitro differentiation toward an osteoclastogenic phenotype [74]. Moreover, in vitro experiments demonstrated that the use of the fully humanized anti-CD38 mAb, daratumumab (DARA), inhibits OC formation and activity, confirming the involvement of CD38 in bone remodeling, in MM patients [74]. CD38 also orchestrates the migration, survival, and Th-1 polarizing ability of mature monocyte-derived dendritic cells through IFN-γ signaling [75]. Moreover, CD38 interacts with the non-substrate ligand CD31, which is constitutively expressed by endothelial cells. Interestingly, a co-expression of CD38 and CD31 was also demonstrated in MM cells but not on PC leukemia [71]. Accordingly, our group has recently reported that extra-medullary MM cells can also lose the expression of CD38 [76]. However, it is not fully known if this effect is a drug-induced microenvironmental change or the selective survival and proliferation of an antigen negative subpopulation. As an ectoenzyme, CD38 represents a metabolic sensor involved in the extracellular conversion of Nicotinamide adenine dinucleotide (NAD)+ to regulators of calcium signaling, such as the immunosuppressive factor adenosine (ADO) [77,78]. This effect occurs through the alternative axis, which includes other ectoenzymes, as CD73 and CD203a, bypassing the canonical pathway mediated by CD39, and it is dependent from the pH status [25]. In line with CD38 strong expression in MM, literature data described higher BM plasma levels of ADO in MM patients as compared with asymptomatic monoclonal gammopathies as MGUS and SMM; moreover, ADO levels correlated with International Staging System (ISS) staging in patients with active disease suggesting that ADO is produced in the MM niche by an ectoenzymatic CD38 network [79]. The source of ADO production in the MM microenvironment has been partially elucidated. Specifically, in vitro data demonstrated that the interactions between MM PCs and other cells of the BM niche, such as OCs, osteoblasts (OBs), and stromal cells induce the production of ADO. Conversely, ADO was not detected in isolated BM microenvironment cells, thus highlighting the role of MM cells in this mechanism [28,29,30]. Recently, our group has also investigated the expression and function of ectoenzymes on microvesicles (MVs) isolated from BM plasma samples of MM patients. Our results showed that the percentage of MVs expressing high levels of ectoenzymes was higher when derived from MM patients compared to MGUS and SMM [80]. The MVs immunophenotype of MM patients indicated a high expression level of CD38, CD39, CD73 and CD203a ectoenzymes as shared by CD138+ PCs. Finally, we demonstrated that the ATP, NAD+, ADPR and AMP to ADO catabolism was higher in MVs from MM patients than in those from controls. This indicates that the ectoenzymes expressed by MVs isolated from BM samples of MM patients were functionally active and involved in the higher ADO production as compared to MGUS and SMM [80]. The peculiar hypoxic and acidic conditions of MM BM niche also support ADO release. Indeed, hypoxia activates the Warburg effect, since aerobic glycolysis represents the main source of cell energy. The consequent accumulation of lactic acid then activates the ectonucleotidases which in turn reduce ATP and increase NAD+, the substrate of the non-canonical CD38/CD203a/CD73 pathway for ADO production. This accumulation in the BM niche then results in an anergic immune status which promotes tumor survival. Indeed, the immune suppressive role of ADO has been deeply elucidated. Among its wide effects [81], ADO impairs DCs ability to prime and amplify Th1 immune responses, in favor of a pro-angiogenic and tolerogenic Th2, by acting on A2b signaling, one of the specific G protein-coupled receptors [82]. Moreover, the engagement of ADO with the other receptor A2a on T cells diminishes their proliferation and secretion of several factors, and induces T cell anergy. A2a activation also blocks the mitogen-activated protein kinase (MAPK) pathway in activated T cell and induces their polarization toward a LAG3+ regulatory phenotype [83,84,85,86]. Interestingly, in vitro studies on murine models revealed that A2a signaling can also upregulate PD-1 expression on both effector and regulatory T cells, thus confirming the immune suppressive role of ADO [87,88]. In light of these observations, new therapeutic strategies targeting ADO-mediated immunosuppression via CD73 and A2a receptor have been designed and entered phase I clinical trials as monotherapy or in combination with PD-1/PD-L1 inhibition in several solid tumors, including Nonsmall-cell lung carcinoma (NSCLC), melanoma, renal cell carcinoma [89,90].

## 7. The Possible Link between CD38 and PD-L1 in MM

Recently it has been reported in preclinical models of different solid tumors that the resistance to anti-PD-1/PD-L1 antibody is mediated by the up-regulation of CD38 by the induction of both all-trans retinoid acid and IFN-β [91]. Authors hypothesized that CD38 expression by cancer cells mediates immune-suppression via ADO production and its effect on CD8+ cytotoxic T cells. Indeed, it was previously reported that inhibition of CD8+ T-cell function by ADO occurs through interaction with ADO receptors ADORA2a and ADORA2b [92]. Studies on mice models demonstrated that CD38-expressing tumor cells impair CD8+ T-cell function and proliferation; however, the treatment with ADO receptor antagonists effectively reversed CD38 suppressive effect on tumor infiltrating CD8+ cells indicating that CD38-mediated production of ADO inhibits CD8+ T-cell proliferation through adenosine receptor signaling [91]. These preclinical data were confirmed in human specimens of lung and melanoma cancers showing a tight relationship between CD38 expression and a cytolytic T cell tumor infiltrate [91]. In addition, a study from Ng HH et al. [93] revealed that CD38 expression on immune cells, especially macrophages, predicts response to PD-1/PD-L1 blocking therapy in patients with hepatocellular carcinoma. Moreover, blocking ADO generation or signaling via CD73 or A2AR, respectively, increased tumor sensitivity to anti–PD-1 therapies [94]. Conversely, ADO also increases PD-1 levels on CD8+ T cells [94]. All together these evidences suggest the existence of a vicious cycle between CD38/ADO and PD-1/PD-L1 axis in cancers; however, this approach has not been explored in depth in MM patients.

**Figure 2:**
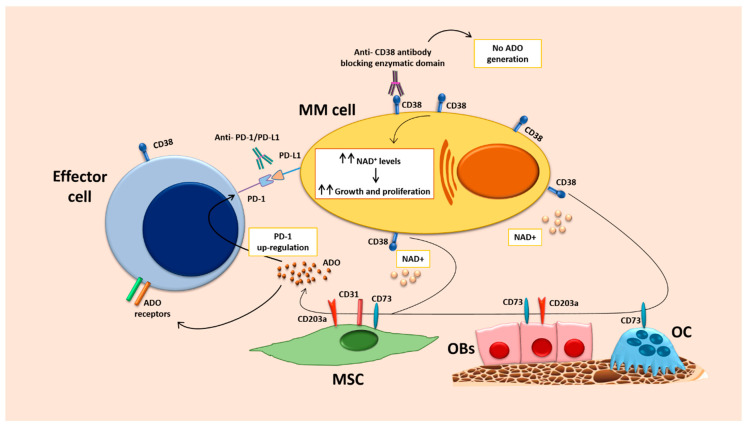
Mechanistic hypothesis behind a synergistic combination between PD-1/PD-L1 blockade and anti-CD38 antibodies inhibiting its enzymatic functions. Abbreviations: ADO, adenosine; MM, multiple myeloma; MSCs, mesenchymal stromal cells; OBs, osteoblasts; OC, osteoclast.

Interestingly, it has been reported that the treatment with anti-CD38 mAb daratumumab (DARA) prevents the increase of PD-L1 expression on antigen presenting cells induced by the standard treatment without DARA in MM patients [95]. Moreover, it has been showed that MM cells increase PD-1 expression by NK and PD-L1 by monocytes and that PD-1/PD-L1 axis suppress the antibody-dependent cellular cytotoxicity (ADCC) mediated by the anti-CD38 mAb isatuximab [96]. Consistently, the combined treatment with isatuximab and anti-PD-L1 or anti-PD-1 antibodies significantly increased the killing of MM cells [96]. Of note, isatuximab but not DARA blocks CD38 enzymatic activity, thus reducing ADO production [97]. It is thus conceivable that isatuximab-mediated ADO reduction and PD-1/PD-L1 blockade could contribute to revert immunesuppression in MM (Figure 2). A role in this scenario could be also played by BM MSCs which exert immune suppressive activities in the MM microenvironment, being involved in ADO release through CD31/CD73/CD203a pathway expressed on their surface and also by promoting MM cell proliferation and T cell inactivation via PD-L1/PD-1 axis [34]. Blocking both CD38 and PD-L1 could thus revert BM MSCs effects and prevent myeloma growth.

Interestingly, a very recent study by Verkleij et al. [98] showed that a long duration treatment with anti-mousePD-1 mAb markedly improved anti-mouse CD38 ADCC in vivo in murine CD38+ myeloma model J558 and other CD38+ tumors.

Overall these data give the rational design to combine the anti-CD38 and the anti-PD-1/PD-L1 blocking antibodies to improve the anti-tumoral effect both in solid tumors and in MM. Ongoing phase I–II trials have been designed in RR-MM patients with the combination of DARA and the anti-PD-1 mAbs, pembrolizumab [99], nivolumab (NCT03184194, NCT01592370) or the anti-PD-L1, durvalumab (FUSION-MM-005) and atezolizumab (NCT02431208). Interestingly, preliminary results from FUSION-MM-005 revealed a low rate of viral reactivation (1 out of 18 patients) as compared to other trials using DARA in monotherapy, which displayed cytomegalovirus and herpes zoster reactivation, mainly due to NK cell depletion [100,101]. These results may suggest that the combination of anti PD-L1/PD-1 mAbs with CD38 blocking Abs could display less toxicity due to infections as compared with the combination with IMiDs; however, only the availability of more data from the ongoing clinical trials could clarify this aspect.

## 8. Conclusions

The discovery of the PD-1/PD-L1 pathway, its role in the evasion of tumor immunity and the development of targeting antibodies represented a great achievement in the immunotherapeutic approach of cancer. Understanding the distribution of PD-1/PD-L1 molecules within BM niche of patients with monoclonal gammopathies and the contribution of the immune resistance mechanisms to PD-1/PD-L1 blockade represents a critical step in order to identify the best subset of patients which could benefit from this checkpoint blockade and to give a rationale for new combined therapeutic strategies. Overall, the majority of the studies published indicate that PD-L1 expression by CD138+ PCs is higher in MM patients as compared to HD subjects and MGUS patients, but they have not found a significant difference between SMM and MM patients or among MM at different stage of disease. Consistently, PD-1 expression seems to be increased in MM patients as compared to HD or MGUS patients. Together with PD-1/PD-L1, many studies indicate that CD38 is involved in the immunosuppression induced by MM cells through the production of ADO [102]. Interestingly, recent data suggest that, among the potential mechanisms behind the lack of responsiveness or resistance to anti-PD-L1/PD-1 antibodies, CD38 metabolic pathways could play an important role. In this regard, blocking CD38 and ADO production could represent an efficient approach to enhance anti-PD-L1 mAbs potential in MM patients. The picture may be completed when information about the signals mediated by the therapeutic anti-CD38 antibodies become available on MM cells and on major effector populations.

## Figures and Tables

**Table 1:** Summary of programmed death-ligand 1/programmed cell death-1 (PD-L1/PD-1) distribution in patients with pre-malignant monoclonal gammopathies and MM at different stages of disease.

PD-L1/PD-1	MM vs. HDs		MM vs. MGUS		MM vs. SMM	MMR vs. MMD		MGUS vs. HDs	Notes	Reference
PD-L1 expression	↑	*p* < 0.01	↑	*p* < 0.01		↑	*p* < 0.021	*p* = NS	CD138+ PD-L1 %	[34]
	↑	*p* < 0.01	↑	*p* < 0.01	-			*p* = NS	CD138+ PD-L1 %	[37]
	↑	*p* < 0.05		-	-		*p* = NS	-	CD138+ PD-L1 %	[39]
	↑	*p* = 0.05 (MRD^+^MM vs HDs)		*p* = NS	-		*p* = NS	*p* = NS	Clonal vs. normal PCs (PD-L1 MFI)	[40]
		*p* = NS		*p* = NS	*p* = NS			*p* = NS	CD138+ PD-L1 %	[41]
	↑	*p* not available							PD-L1 MFI on CD138+	[38]
		*p* = NS	↑	*p* = 0.03	*p* = NS		*p* = NS	-	PD-L1 MFI on CD138+	[46]
		-		*p* = NS	*p* = NS		*p* = NS	-	PD-L1 MFI on CD14+	[46]
	↑	*p* not available (MM BM vs. HD PB)		-	-		-		PD-L1 MFI on DCs	[48]
PD-1 expression		*p* = NS		-	-		*p* = NS	-	CD4+ PD-1%	[39]
	↑	*p* < 0.05		-	-		*p* = NS	-	CD8+ PD-1%	
		*p* = NS	↑	*p* < 0.05 (MGUS+MMD+ MRD^−^ vs. MRD^+^)	-		*p* = NS	*p* = NS	CD4+ and CD8+ PD-1 %	[40]
	↑	*p* not available		-	-		-	-	PD-1 MFI on total T cells	[38]
	↑	*p* < 0.05		-	-		*p* = NS	-	CD8+ PD-1%	[55]
		-		*p* = NS	*p* = NS		*p* = NS	-	CD4+ and CD8+ PD-1 %	[46]

Abbreviations: PCs, plasmacells; NS, not significant; MGUS, monoclonal gammopathy of undetermined significance, HDs, healthy donors; MM, multiple myeloma; SMM, smoldering MM; MMD, newly diagnosed MM; MMR, relapsed MM; MRD, minimal residual disease; BM, bone marrow; PB, peripheral blood; DCs, dendritic cells; arrow, up-regulated; -, not evaluated.

**Table 2:** PD-L1/PD-1 regulation in MM niche by immunomodulatory drugs (IMiDs).

IMiD	MM Cells	T Cells	NK Cells	Monocytes/ MDSCs	Ref
Lenalidomide	PD-L1 down-regulated	PD-1 down-regulated	PD-1 down-regulated	PD-L1 down-regulated	[39]
	PD-L1 up-regulated in IMiDs resistant cells				[64]
Pomalidomide	PD-L1 down-regulated	-	-	PD-L1 down-regulated	[65]
	PD-L1 up-regulated in IMiDs resistant cells				[64]
Thalidomide	-	-	-	-	

Abbreviations: MDSCs, myeloid derived suppressor cells; MM, multiple myeloma; NK, natural killer; Ref, references; -, data not available.

## Data Availability

Not applicable.
